# Quantitative Contrast-Enhanced Magnetic Resonance Lymphangiography of the Upper Limbs in Breast Cancer Related Lymphedema: An Exploratory Study

**DOI:** 10.1089/lrb.2014.0039

**Published:** 2015-06-01

**Authors:** Marco Borri, Maria A. Schmidt, Kristiana D. Gordon, Toni A. Wallace, Julie C. Hughes, Erica D. Scurr, Dow-Mu Koh, Martin O. Leach, Peter S. Mortimer

**Affiliations:** ^1^ CR-UK Cancer Imaging Centre, Institute of Cancer Research and Royal Marsden NHS Foundation Trust, Sutton, United Kingdom.; ^2^ Skin Unit, Royal Marsden NHS Foundation Trust. Sutton, United Kingdom.; ^3^ St. George's, University of London, Cardiac and Vascular Sciences, London, United Kingdom.

## Abstract

***Background:*** Contrast-Enhanced Magnetic Resonance Lymphangiography (CE-MRL) presents some limitations: (i) it does not quantify lymphatic functionality; and (ii) enhancement of vascular structures may confound image interpretation. Furthermore, although CE-MRL is well described in the published literature for the lower limbs, there is a paucity of data with regards to its use in the upper limbs. In this proof-of-principle study, we propose a new protocol to perform CE-MRL in the upper limbs of patients with breast cancer-related lymphedema (BCRL) which addresses these limitations.

***Methods and Results:*** CE-MRL was performed using a previously published (*morphological*) protocol and the proposed protocol (*quantitative*) on both the ipsilateral (abnormal) and contralateral (normal) arms of patients with BCRL. The quantitative protocol employs contrast agent (CA) intradermal injections at a lower concentration to prevent T2*-related signal decay. Both protocols provided high-resolution three-dimensional images of upper limb lymphatic vessels. CA uptake curves were utilized to distinguish between lymphatic vessels and vascular structures. The quantitative protocol minimized venous enhancement and avoided spurious delays in lymphatic enhancement due to short T2* values, enabling correct CA uptake characterization. The quantitative protocol was therefore employed to measure the lymphatic fluid velocity, which demonstrated functional differences between abnormal and normal arms. The velocity values were in agreement with previously reported lymphoscintigraphy and near infra-red lymphangiography measurements.

***Conclusions:*** This work demonstrated the feasibility of CE-MRL of the upper limbs in patients with BRCL, introducing an advanced imaging and analysis protocol suitable for anatomical and functional study of the lymphatic system.

## Introduction

Breast Cancer-Related Lymphedema (BCRL) remains one of the most common and distressing morbidities in breast cancer survivors treated with radical surgery and axillary nodal dissection.^1^ Despite advances in breast cancer treatment, the prevalence of BCRL remains high at more than 20%.^2^ However, clinical imaging of the lymphatic system is limited: direct contrast lymphography is no longer practiced as it is invasive and risks exacerbating the lymphedema.^3^ Lymphoscintigraphy is currently the most widely utilized investigation for evaluating lymphedema, but suffers from poor spatial resolution.^4^ Near infra-red lymphangiography using indocyanine green is a recently introduced technique with high spatial resolution, but demonstrates only superficial lymphatic vessels.^5^

High resolution contrast-enhanced magnetic resonance lymphangiography (CE-MRL) has been applied to study deep and superficial lymphatic vessels in the lower limbs.^6,7^ It requires an interstitial injection of gadolinium-based paramagnetic contrast agent (CA), which is taken up by the lymphatic system, leading to lymphatic enhancement on T1-weighted images. The ideal paramagnetic contrast agent for CE-MRL would be a lymphotrophic molecule large enough to be taken up selectively in the lymphatic system. However, the conventional paramagnetic contrast agents currently available are relatively small molecules, and are also taken up in the cardiovascular system when an interstitial injection is administered. Enhancement of vascular structures may therefore confound image interpretation.

Although CE-MRL of the lower limbs is well described in the published literature, there is a paucity of data with regards to its use in the upper limbs. In this work we (1) demonstrate CE-MRL of the upper limbs in patients with BRCL; (2) employ the CA uptake dynamic information to distinguish lymphatic vessels from blood vessels; (3) reduce the CA concentration at the injection site to enable quantitative analysis of the images, defining a protocol where signal intensity is proportional to the CA concentration within the lymphatic vessels; (4) quantify lymphatic transport within the main lymphatic vessels in order to provide an objective measure of the degree of lymphatic dysfunction.

## Materials and Methods

The study was approved by the institutional research and ethics committee. Written informed consent was obtained from all patients prior to inclusion in the study.

### Clinical examinations

For this proof-of-principle study, 3 female patients with unilateral BCRL were recruited. The age of the patients ranged from 49 to 61 years, the average weight was 73.3 kg, and none of the patients had contraindications to MRI or contrast agents. Both the ipsilateral (affected) and contralateral (unaffected) arm were imaged at 1.5 T (MAGNETOM Aera, Siemens AG, Erlangen, Germany), on separate visits. The patient was positioned supine, with the examined arm extended along the body; the torso was then rotated by 45°, bringing the arm towards the center of the magnet.

The imaging protocol included a high spatial resolution three-dimensional (3D) T1-weighted fast-spoiled gradient-echo pulse sequence, with parameters sensitive to contrast enhancement: TE/TR=2.77/6.14 ms, flip angle=12°, voxel size=1×1×1 mm, spectrally selective fat suppression. The whole arm was imaged in three stations, covering the anatomy from the hand to the axilla with three partially overlapping volumes. For each station, a volume with FOV=300 mm in the superior/inferior direction was acquired in 1 min and 18 sec. The total volume (total acquisition time=3 min and 54 sec) was imaged once pre-injection and several times post-injection over a period of 45 minutes. A mixture of gadoteridol (ProHance®, Bracco Diagnostics Inc., Princeton, USA, [Gd]=0.5 M) and anesthetic (1% lidocaine) was administered with a 1 mL total volume intradermal injection for each of the four inter-digital spaces. Two different injection protocols were adopted:

*Morphological* CE-MRL Protocol: 1 mL of injected volume contains 0.9 mL of gadoteridol and 0.1 mL of anesthetic,^6^ with resulting [Gd]=0.45 M.

*Quantitative* CE-MRL Protocol: 1 mL of injected volume contains 0.02 mL of gadoteridol, 0.1 mL of anesthetic and 0.88 mL of saline, with resulting [Gd]=0.01 M.

Patient 1 and 2 were examined with the *morphological* protocol (both arms). The ipsilateral (affected) arm of Patient 2 was also examined with the *quantitative* protocol. Patient 3 was examined with the *quantitative* protocol (both arms).

### Evaluation of relaxation parameters

In spoiled gradient-echo sequences with short TE commonly employed in CE-MRL, short T2* values should be avoided to ensure that T2* signal decay is negligible. We measured T1 and T2* in a series of progressive dilutions of the initial contrast solution adopted in the morphological protocol (Solutions A–D, Table 1), using a double flip angle method and in-house software.^8^ This information was used to identify the optimal contrast dilution for quantitative CE-MRL.

**Table 1.** Measurement of Relaxation Parameters

	*Morphological protocol*	*Solution A*	*Solution B*	*Solution C*	*Solution D*
Gadoteridol (mL)	0.900	0.060	0.030	0.015	0.006
1% lidocaine (mL)	0.100	0.100	0.050	0.025	0.010
Saline, 0.9% NaClO_3_ (mL)	None	0.840	0.920	0.965	0.994
[Gd] (M)	0.4500	0.0300	0.01500	0.0075	0.0030
T2^*^ (ms)	−†	6.3±0.1	8.8±0.1	13.1±0.2	24.8±0.1
T1 (ms)	−†	14±2	20±3	19±3	35±5

T2^*^ and T1 (average±standard deviation) in solutions of gadoteridol (ProHance®) and anesthetic (1% lidocaine) progressively diluted in saline. Volumes are provided per 1 mL injection. [Gd]=concentration of gadolinium (M); †indicates values too short to be measured by standard MRI sequences (T2^*^ <2 ms, T1 <10 ms).

Physiological modeling of the exchange between the depot created by the injection and the venous system (Appendix 1) allowed estimation of the peak T1 of enhanced venous blood immediately following the interstitial injection (T1_post_).

### Image analysis

Image processing was performed with in-house software developed in IDL (version 8.2, Exelis Visual Information Solutions, Boulder, Colorado, USA). Each post-contrast volume was subtracted from the first volume with the purpose of visualizing the CA uptake. Prior image registration across the dynamic series was required to compensate for patient motion and to avoid subtraction artifacts. This was performed by incorporating a previously reported 3D rigid body registration package^9^ into the software. The volume imaged at each station (hand, arm, or axilla) was then registered, assuming that each station would experience mainly rigid motion. The software was designed to visualize the entire subtracted 3D data volume, producing, for each time point, a maximum intensity projection (MIP) along a defined direction. Each voxel in the MIP was associated with the corresponding dynamic uptake curve, which plots the evolution of the signal with time. The uptake curves were fitted with a five-parameter modified logistic equation^10^ (see Fig. 3). This enabled identification of the onset time (time of arrival of the CA) and characterization of the shape of the curve with five numerical parameters: P1 represents the baseline signal, P2 the net enhancement, P3 the time of contrast onset, P4 the maximum slope (onset slope), and P5 the terminal slope. This semi-quantitative heuristic model is general, and has been used to describe a wide range of signal enhancement patterns.^10^

The developed image processing tools were used to (a) measure the image intensity of the depot for both the *morphological* and the *quantitative* protocols, (b) measure the image intensity of enhancing structures for both injection protocols, (c) differentiate the enhancement of veins from that of lymphatic vessels, and (d) measure the lymphatic fluid velocity in the ipsilateral (affected) and the contralateral (unaffected) arms imaged with the quantitative protocol. The fluid velocity was calculated for a determined section of lymphatic vessel as the ratio between the distance travelled and the difference in onset of enhancement between the two extremities of the section.

## Results

### Evaluation of relaxation parameters

Table 1 shows T1 and T2* values for CA and anesthetic solutions. The T2* of the solution employed in the morphological protocol was too short to be measured with standard MRI sequences. T2* decay will therefore dominate early characterization of enhancement and T2*-related signal loss will remain significant until further dilution takes place. The CA concentration ([Gd]) adopted in the quantitative protocol lies between Solution B and Solution C, and therefore 8.8 <T2* <13.1 ms. This corresponds to a percentage signal decay no larger than 26% (derived from the exponential term e^-TE/T2*^). Further dilution in the depot and in the lymphatic system will then result in a negligible T2* decay in all the images acquired with the quantitative protocol.

The T1_post_ values of venous blood estimated by the physiological modeling (Appendix 1) were 697 ms and 1183 ms for the morphological and quantitative protocols, respectively, indicating that the T1 shortening (T1_pre_−T1_post_, T1_pre_=1200 ms) should produce an appreciable signal enhancement of venous blood only with the morphological protocol. Enhancement of veins should therefore be negligible in all the examinations performed with the *quantitative* protocol.

### Image analysis

All the examinations were reviewed by a radiologist together with the clinical scientists to determine the presence and extent of lymphatic enhancement.

**FIG. 1.**
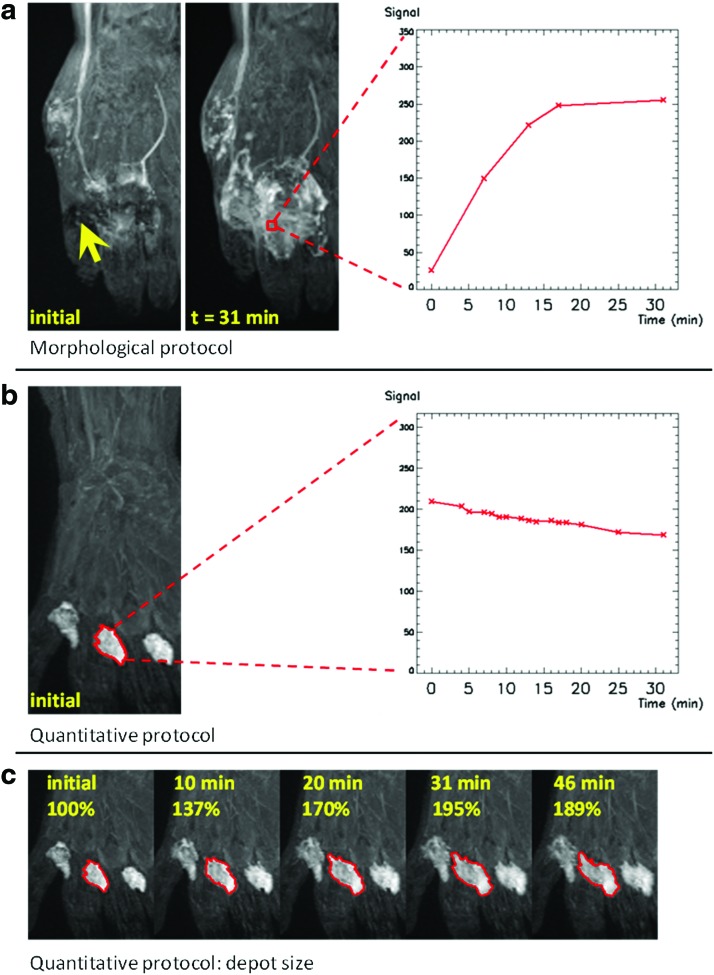
Evolution of the depot (hand) for the two different protocols. Images are coronal maximum intensity projections (MIP) of the original image volumes. **(a)** Morphological protocol (Patient 1, 49-year-old, female, ipsilateral arm). In the image acquired immediately after the injection, the depot area is affected by signal loss due to short T2* effects (*yellow arrow*). The graph plots the evolution of the signal with time, in a voxel within the depot area (*red*) selected on a later MIP. **(b)** Quantitative protocol (Patient 3, 50-year-old, female, ipsilateral arm). The region of interest (ROI, *red*) is contoured on the initial MIP and encompasses one of the four inter-digital depots. The graph shows the decrease with time of the mean signal from the ROI. **(c)** Expansion with time of the projected depot delineated in **(b)**. A color version of this figure is available in the online article at www.liebertpub.com/lrb.

With the morphological protocol (an example in Fig. 1a), it is not possible to delineate the initial depot as the injection area is non-uniformly affected by signal loss due to short T2* values (indicated by the arrow). In this area, the signal increases with time, as shown in the graph, despite the fact that the concentration of CA decreases. Progressive removal of CA results in dilution in the depot, increasing the T2* and reducing the T2*-related signal decay. In examinations undertaken with the quantitative protocol (an example in Fig. 1b), the depot area could be clearly identified in all images, indicating that T2* decay has been eliminated. The evolution of the depot with time (Fig. 1b and 1c) indicates slow removal of CA, in agreement with the expected physiological behavior.^4^

**FIG. 2.**
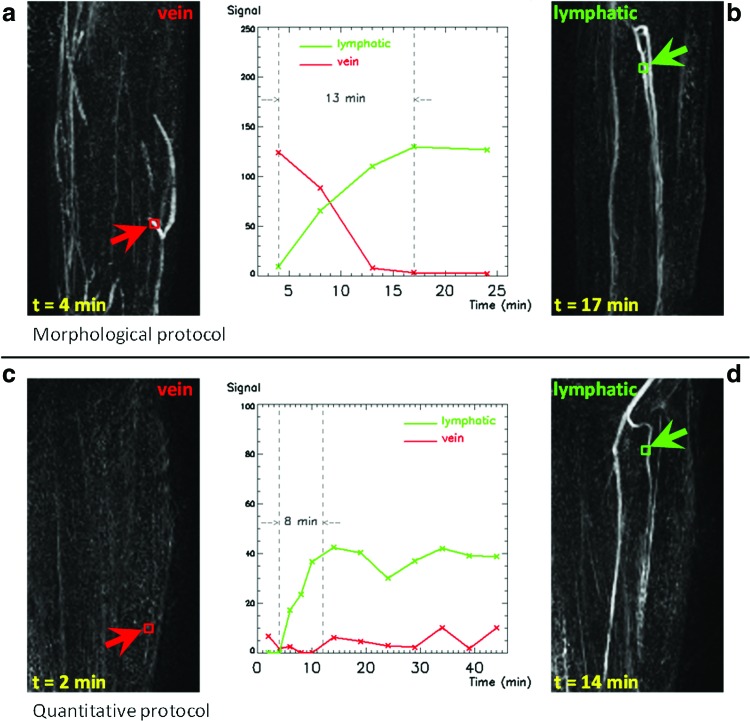
Enhancement of lymphatic vessels and veins in the forearm of Patient 2 (61-year-old, female, ipsilateral arm), for the two different protocols, and associated graphs plotting the evolution of the signal with time, including a measurement of rising time for lymphatic enhancement. Voxels belonging to the same structures are selected for both protocols; *green color* refers to lymphatic vessels, *red* to veins. Coronal maximum intensity projections (MIP) are created from image volumes at different time points, after subtracting the first post-contrast volume in order to visualize the evolution of the enhancement from the baseline. Veins are not visible in images acquired with the quantitative protocol. Lymphatic vessels have a later and slower enhancement compared with veins. The same lymphatic vessels can be identified with both protocols. **(a, b)** Morphological protocol: veins, lymphatic vessels; **(c, d)** Quantitative protocol: veins, lymphatic vessels. A color version of this figure is available in the online article at www.liebertpub.com/lrb.

Figure 2 compares the examinations performed with both protocols on the affected arm of Patient 2, showing images of enhancing vessels. The pattern of enhancement of the vessel, described by the CA uptake curve, can be used to discriminate between lymphatic vessels and veins. Veins show initial signal enhancement and subsequent signal decay due to CA wash-out (red in the plot, Fig. 2a), whereas structures that slowly take up contrast and appear bright at a later time are lymphatic vessels (green in the plot, Fig. 2b and 2d). Despite a slight difference in arm position in different examinations, the same lymphatic vessels can be identified with both protocols (Fig. 2b and 2d). In general, the enhancement of veins is negligible in all the images acquired with the quantitative protocol (an example in Fig. 2c), as predicted by the physiological modeling (Appendix 1), and the lymphatic vessels are therefore the only visible structures in these images.

Figure 2 also suggests that T2* decay is significant within the lymphatic vessels imaged with the morphological protocol: the slower rise in image intensity of the lymphatic vessels (13 min, Fig. 2b) is likely to be associated with a progressive lengthening of the T2* by further dilution of CA (similar to the effect observed in the depot). The dynamic curves obtained with the quantitative protocol are not affected by T2* decay and have a shorter time to peak (Fig. 2d).

**FIG. 3.**
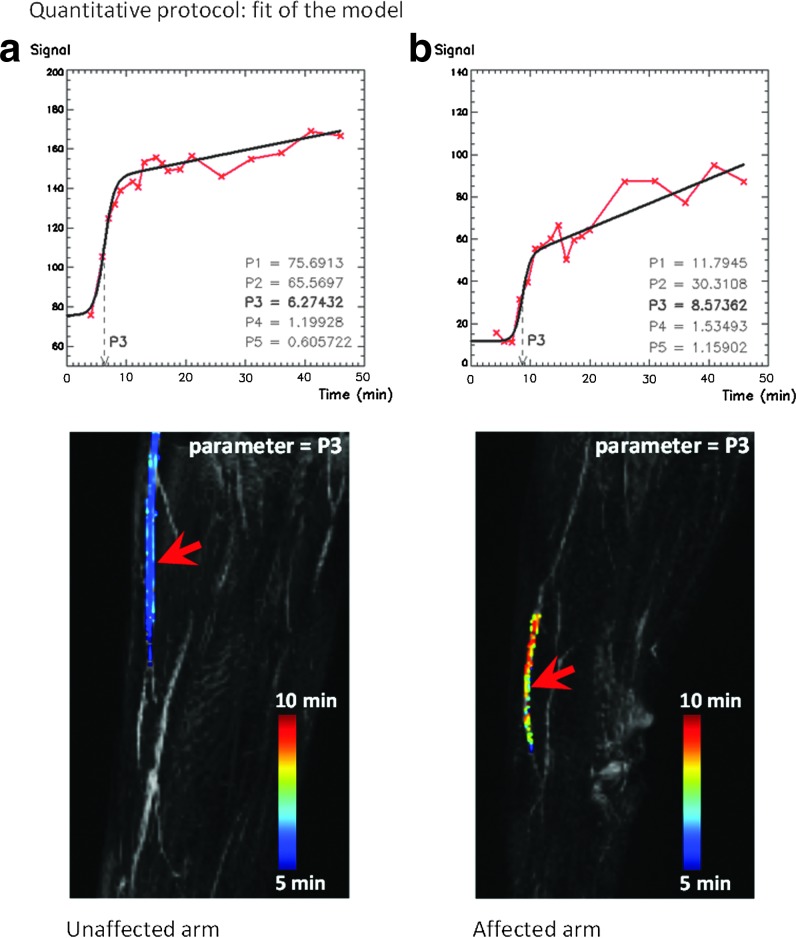
Five-parameter logistic model^10^ applied to images acquired with the quantitative protocol (Patient 3, 50-year-old, female) and employed to compare the lymphatic fluid velocity between the unaffected (contralateral) and the affected (ipsilateral) arm. The plots display the signal from the selected voxels (*red arrows*). The curve is fitted with a modified logistic equation (*black line*). The five parameters (P1–P5)^10^ describe the shape and the timing of the curve. **(a)** Distribution of the parameter P3 (onset time, minutes) within a main lymphatic vessel in the contralateral arm, superimposed, with color scaling, on the maximum intensity projection (MIP). The difference in onset along the lymphatic vessel indicates that the contrast agent is perfusing the vessel. **(b)** Distribution of the parameter P3 within a main lymphatic vessel in the arm with impaired lymphatic function (ipsilateral), demonstrating a slower CA uptake.

Figure 3 shows a similar section of the affected and unaffected arms of Patient 3, imaged with the quantitative protocol. In Figure 3b and 3c, the increase in onset time (parameter P3) along the lymphatic vessel demonstrates that the CA is perfusing the vessel, with slower progression in the arm with impaired lymphatic function. The delay in onset between a proximal and a distal point in the vessel represents the time that the enhanced fluid employs to travel along the vessel. The velocity of the lymphatic fluid can therefore be calculated as the distance between these two points divided by the difference in P3. The velocity was found to be 9.7 cm min^−1^ for the contralateral (unaffected) arm, and 2.1 cm min^−1^ in the ipsilateral (affected) arm.

## Conclusions

In this article we present high resolution (1 mm isotropic) MR images of lymphatic vessels in the upper limbs at 1.5 T. The current CE-MRL literature focuses on lower limbs and reports the use of interstitial injections of CA at high concentration without discussing the potentially confounding effects of T2* relaxation.^6,7^ Improvement resulting from a diluted injection, administered to avoid adverse reactions to the CA, was previously reported,^11^ but this was not discussed or associated with T2* relaxation.

In this work we provide evidence of T2*-related effects in images acquired following injections of CA at high concentration. Current CE-MRL protocols employ parameters suited to quantitative dynamic contrast-enhanced (DCE) examinations performed with intravenous CA injection. However, in DCE-MRI T2* decay can be neglected, due to the substantial CA dilution in the blood system. In CE-MRL examinations, although the CA experiences a further dilution in the lymphatic vessels, this is not expected to be considerable: this is supported by the observation that the peak signal in the depot (Fig. 1b) and in the main lymphatic vessels (Fig. 2d) are of the same order of magnitude. Therefore, in order to avoid T2* effects, it is necessary to reduce the CA concentration.

Ideally, one should optimize the CA concentration in order to both avoid T2* effects and to obtain the maximum signal-to-noise ratio (SNR) within the lymphatic vessels. In this study we conservatively optimized the CA concentration at the site of injection (highest CA concentration). Accurate assessment of the extent of further dilution in the lymphatic vessels requires a measurement of the concentration of gadolinium within the vessel. This could be derived from in vivo measurements of T1 of the lymphatic fluid pre and post injection, and the proposed quantitative protocol, being unaffected by T2*-related relaxation, would be suitable for such measurements. In future, this could lead to further optimization of both the CA concentration of the injection and of the MRI sequence parameters, and could maximize the signal within the lymphatic vessels. However, whilst the quantitative protocol was not designed to maximize the SNR of the lymphatic vessels, the resulting images exhibited the same lymphatic structures as the ones from the morphological protocol, despite being produced with a significantly lower CA concentration.

Our research demonstrates the importance of optimizing CE-MRL protocols, considering the relaxation parameters involved in signal enhancement. T2* decay results in signal loss and spurious delay in enhancement, and introduces a nonlinear relationship between the signal intensity and the concentration of CA, which could potentially bias velocity measurements dependent on progression of enhancement. The proposed quantitative protocol produces images where the CA concentration is proportional to the image intensity and therefore enables quantitative analysis of the images. With this protocol it was possible to expand the analysis of lymphatic enhancement by fitting a logistic equation^10^ to the uptake curve. We successfully employed the onset time (parameter P3) to demonstrate the progression of enhancement along the main lymphatic trunk of the arm and to calculate the lymphatic fluid velocity. The obtained values are in agreement with the estimates reported using lymphoscintigraphy (8.9±5.8 and 3.2±8.9 cm min^−1^ for unaffected and affected arms, respectively)^12^ and near infra-red lymphangiography (10.0±3.38 and 2.38±1.08 cm min^−1^ for unaffected and affected arms, respectively).^5^

Differences in uptake and lymphatic fluid velocity are ultimately expected to produce variations in all the model parameters. We limited our evaluation to the parameter P3 (onset time) because its progression along the vessel is directly related to the fluid velocity, while differences in signal depend on differences in CA concentration and dilution patterns within the vessels. However, one could infer that the curve in the affected arm (Fig. 3b) has a lower net enhancement (P2) and a slower rise (P4–P5); this is compatible with a slower uptake and impeded transport ability. In future work, larger cohorts and CA concentration measurements will enable investigation of differences in all the parameters of the model.

The CA uptake curve describes CA transport in the vessel and therefore characterizes the underlying physiology. Lymphatic structures can be depicted and distinguished from vascular structures as the lymphatic uptake curve has a distinctive shape resulting from the CA moving at relatively slow speed in the vessel. The dynamic information contained in the CA uptake curve is the key feature which transforms classic CE-MRL into a functional technique for the first time. The proposed quantitative protocol enables correct characterization of the CA uptake curve, allowing quantification of lymphatic transport. This has the potential to track the effects of therapy and to stratify patients for different treatments.^13,14^ In future work, further analysis of the CA uptake curves could provide a more complete description of lymphatic functionality.

We acknowledge that our study is limited to seven examinations conducted on 3 patients with BCRL. However, even with this small sample, we have addressed fundamental issues affecting CE-MRL. Quantitative CE-MRL has the potential to expand the imaging of the lymphatic system, and overcome the limits imposed by other techniques. This approach now warrants evaluation in a larger cohort of patients.

In conclusion, we have employed CE-MRL to produce high resolution MR images of upper limb lymphatic vessels at 1.5 T. We showed that the pattern of CA uptake curves can discriminate between enhancing lymphatic vessel and veins. Furthermore, we proposed a new quantitative protocol, which minimized venous enhancement and prevented T2*-related signal loss, allowing correct CA uptake modeling and measurement of lymphatic fluid velocity. The proposed imaging and analysis protocol is suitable for quantitative studies and can be used for both structural and functional evaluation of the lymphatic system within the same examination.

## Appendix 1 (Estimation of Contrast Agent Concentration in Venous Blood)

An interstitial injection of contrast agent creates a depot in the webspace of the hand. For this study we used gadoteridol, a hydrophilic molecule of molecular mass 558.7 g mol^−1^, confined to extracellular water, and therefore subjected to a diffusion-limited transcapillary exchange.^15^ The initial end-capillary concentration of CA within the depot (C_cap_) will be lower than the concentration in the depot, and will fall with time as CA is removed by both the lymphatic and the venous systems. Consequently, the injected depot concentration can be considered the upper limit for C_cap_. The ratio of the local plasma flow through the depot to the plasma flow through the arm (F_ratio_) represents the minimum dilution of C_cap_ in the venous system of the whole arm. F_ratio_ can be approximated as the ratio of the tissue accessed in the depot to the total tissue in the arm. Assuming that 1 mL of solution accesses to the microcirculation in at most 1 g of tissue of a 3.3 kg arm (approximately 4.7% of the total body mass of a 70 kg person^16^), F_ratio_=1 g/3300 g=0.0003. Considering four inter-digital depots, the initial CA concentration in venous blood in the arm should not exceed [Gd]_veins_=4*0.0003*C_cap_.

If the maximum possible C_cap_ is 0.45 M and 0.01 M for morphological and quantitative protocols, respectively, assuming unenhanced blood (T1_pre_=1200 ms at 1.5 T) and using the formula:^17^

\begin{align*}1 / {\rm T1}_{\rm post} = 1 / {\rm T1}_{\rm pre} +
r * [ {\rm Gd} ] _{\rm veins}\end{align*}

where r=4.4 mM^−1^ s^−1^ is the relaxivity of gadoteridol,^18^ the peak T1 of enhanced venous blood (T1_post_) is calculated as 697 ms and 1183 ms for the morphological and quantitative protocols, respectively.
